# Regulation of Gene Expression under Hypoxic Conditions

**DOI:** 10.3390/ijms20133278

**Published:** 2019-07-03

**Authors:** Koh Nakayama, Naoyuki Kataoka

**Affiliations:** 1 Oxygen Biology Laboratory, Medical Research Institute, Tokyo Medical and Dental University (TMDU), Tokyo 113-8510, Japan; 2 Laboratory of Cell Regulation, Departments of Applied Animal Sciences and Applied Biological Chemistry, Graduate School of Agriculture and Life Sciences, The University of Tokyo, Tokyo 113-8657, Japan

**Keywords:** Hypoxia, transcription, HIFs, Splicing, SR proteins, SR protein kinases

## Abstract

Eukaryotes are often subjected to different kinds of stress. In order to adjust to such circumstances, eukaryotes activate stress–response pathways and regulate gene expression. Eukaryotic gene expression consists of many different steps, including transcription, RNA processing, RNA transport, and translation. In this review article, we focus on both transcriptional and post-transcriptional regulations of gene expression under hypoxic conditions. In the first part of the review, transcriptional regulations mediated by various transcription factors including Hypoxia-Inducible Factors (HIFs) are described. In the second part, we present RNA splicing regulations under hypoxic conditions, which are mediated by splicing factors and their kinases. This work summarizes and discusses the emerging studies of those two gene expression machineries under hypoxic conditions.

## 1. Introduction

The earth’s atmosphere contains 20.9% oxygen, and, consequently, most living creatures are exposed to this element. Mitochondria use oxygen to efficiently produce energy, which is beneficial for cells. On the other hand, oxygen can cause oxidative stress, which damages cells and induces cell death. Therefore, organisms have developed multiple strategies to cope with different oxygen environments. One such strategy is the hypoxic response, which is triggered upon exposure of cells to a low oxygen environment. Hypoxic conditions arise in tissues because oxygen is not freely available throughout the body. This environment, in which a moderately low oxygen concentration is maintained in comparison with the atmosphere (called physiological normoxia), reflects the normal oxygen status in each tissue and cell [1]. By contrast, at high altitude and under pathological conditions such as ischemia, cells and tissues are deprived of oxygen, which results in severely hypoxic conditions. Organisms must respond properly to such conditions in order to survive. The hypoxic response is a systemic process that regulates multiple cellular activities to maintain homeostasis under hypoxic conditions [2]. This response enhances oxygen delivery by increasing the number of red blood cells or blood vessels, alters energy metabolism, increases cell motility, and thereby enables cell adaptation and prevents cell death. The hypoxic response protects against stresses, but promotes disease progression in some pathological conditions. Hypoxic conditions arise in ischemic diseases and conditions, such as stroke, heart attack, cancers, and inflammatory diseases. The hypoxic response facilitates survival of cancer cells and induces their invasion and metastasis, leading to a poor prognosis [3].

Oxygen levels are sensed by different mechanisms in the human body. Humans possess a structure called the carotid body, which is located in the carotid artery that senses oxygen and carbon dioxide levels in blood. Once the carotid body detects a decrease in the blood oxygen level, it becomes excited and transduces a signal to stimulate breathing, thereby increasing the acquisition of oxygen from the atmosphere [4]. A family of enzymes called 2-oxoglutarate (2-OG)-dependent oxygenases [5] require oxygen for their activity and are inhibited when oxygen becomes limited. One of the best-characterized 2-OG-dependent oxygenases is prolyl-hydroxylase PHD, that negatively regulates Hypoxia-Inducible Factor (HIF) (described in Section 2). In principle, these enzymes sense changes in the oxygen level by altering their enzymatic activities. Specifically, they are active in the presence of oxygen and less active in the absence of oxygen.

**Figure 1:**
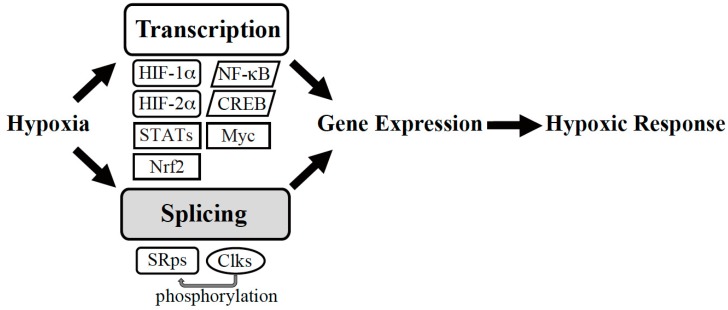
Gene expression machinery under hypoxia. Hypoxic conditions trigger transcription and splicing to induce expression of gene sets required for hypoxic adaptation. HIF: Hypoxia-Inducible Factor, NF-κB: nuclear factor-kappa B, CREB: cAMP-response Element-binding Protein, Nrf: NF-E2-related factor, STATs: signal transducers and activators of transcription, SRps: serine-arginine-rich (SR) proteins, Clks, Cdc2-like kinases.

In general, hypoxic conditions are energetically challenging for cells because oxygen becomes a limiting factor and, consequently, mitochondrial respiration decreases. Cells respond by inhibiting energy-consuming processes to conserve energy. One of the most energy-consuming processes in cells is protein translation. Thus, protein synthesis is significantly inhibited under hypoxic conditions [6]. This is partly mediated by reduced phosphorylation of 4E-BP1, which is an important regulator of protein translation [7,8]. There are also effects on transcription (described in Section 2) and splicing (described in Section 3); some transcription and splicing factors are activated under hypoxic conditions (Figure 1). These machineries are described in this review.

## 2. Transcriptional Regulation under Hypoxia

### 2.1. Hypoxia-Inducible Factor (HIF)

**Figure 2:**
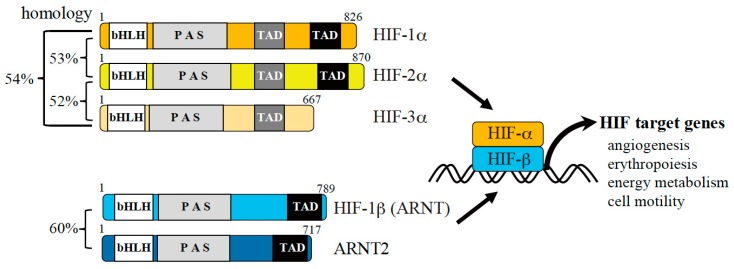
Hypoxic gene expression mediated by hypoxia-inducible factors (HIFs). There are three α and two β subunits of HIFs (numbers indicate the lengths of the amino acid sequences corresponding to human proteins). The α subunit is regulated by oxygen, while the β subunit is constitutively expressed. The α subunit is stabilized under hypoxic conditions and forms a heterodimer with the β subunit, leading to transactivation of HIF target genes. The target genes of HIF regulate angiogenesis, erythropoiesis, energy metabolism, cell motility, and other processes to promote hypoxic adaptation of cells.

Some transcription factors are activated under hypoxic conditions. Among such factors, HIF is a central player that regulates hypoxic responses. This factor consists of α and β subunits. The α subunit of HIF (HIF-α) was originally identified as a nuclear factor that binds to the 3′ enhancer region of the *EPO* gene [9]. There are three α subunits (HIF-1α, HIF-2α, and HIF-3α) and two β subunits (HIF-β and ARNT2) of HIF. These subunits have a similar domain structure and belong to the bHLH-PAS protein family, although homology at the amino acid level is only around 50% for HIF-1α and HIF-2α. HIF-α and HIF-β form a heterodimer in the nucleus, and induce expression of multiple genes (Figure 2). More than 100 genes have been identified as targets of HIF-1 [10]. These genes can be categorized according to their biological functions, such as metabolism, angiogenesis, anti-apoptosis, and cell motility. These biological processes are important for proper adaptation of cells to hypoxia. Among the various HIF subunits, HIF-1α and HIF-2α have been extensively studied. These two subunits have many overlapping roles as well as several different functions. One important characteristic of HIF is that expression of the α subunit is regulated in an oxygen-dependent manner [11]. Under normoxic conditions in which oxygen is freely available, HIF-α is degraded by the ubiquitin-proteasome pathway [12,13]. By contrast, under hypoxic conditions, in which oxygen is limited, HIF-α is stabilized. Owing to its oxygen-dependent expression and activation, HIF-α was thought to sense oxygen. Biochemical analyses revealed that HIF-α contains an oxygen-dependent degradation domain, which plays a critical role in oxygen-dependent expression of this subunit [14,15]. Subsequent analyses demonstrated that the oxygen-dependent degradation domain contains proline residues, which are critical for this oxygen dependency. These proline residues are hydroxylated and determine the fate of HIF-α [16,17]. Prolyl hydroxylase domain-containing protein (PHD) is an enzyme that hydroxylates the proline residues of HIF-α [18]. It belongs to the 2-OG-dependent oxygenase family and requires co-factors such as Fe^2+^, 2-OG, and oxygen for its activity. Because it requires oxygen as a co-factor, PHD functions as an oxygen-sensing molecule [19]. When HIF-α is prolyl-hydroxylated, it efficiently binds to pVHL, a subunit of the ubiquitin ligase complex [13,20]. pVHL forms an active ubiquitin ligase complex together with elongin B, elongin C, Cullin2, and Rbx1, and ubiquitinates HIF-α in normoxia [21]. *pVHL* is a causative gene of von Hippel-Lindau (VHL) disease. Certain types of pVHL mutation cause renal cell carcinoma, hemangioblastoma, and pheochromocytoma in this disease, whereas another type of pVHL mutation causes a hematological disorder called Chuvash polycythemia, which does not increase the risk of developing cancer [22,23]. HIF-α is aberrantly stabilized in these patients. There is another type of 2-OG-dependent oxygenase called Factor Inhibiting HIF (FIH). FIH also hydroxylates HIF on the asparagine residue, and suppresses transcriptional activity of HIF by outcompeting p300 [24]. Mice with knockout (KO) of HIF-1α and HIF-2α have been generated. The absence of HIF-1α or HIF-2α causes embryonic lethality, indicating that the functions of these two subunits are critical for proper development [25,26,27,28]. Furthermore, analyses of conditional HIF-KO mice revealed the roles of these factors in physiological and pathological conditions, including immune responses [29].

### 2.2. NF-κB

While HIF has been extensively studied as a key molecule for regulation of gene expression during the hypoxic response, many other transcription factors also function under hypoxic conditions. NF-κB is one such factor. The role of NF-κB is well-characterized in immune cells that mediate inflammatory responses [30]. The NF-κB family consists of five members, and the canonical signaling is mainly mediated by p50-p65 dimers [31]. NF-κB is activated during the early phase of hypoxia in macrophages and pulmonary artery smooth muscle cells [32,33]. In monocytes, activated NF-κB binds to the promoter region of HIF-1α and thereby induces its expression [34]. Interestingly, HIF-1 induces expression of NF-κB in neutrophils [35]. Thus, there appears to be a positive regulatory loop between NF-κB and HIF-1, although regulation of these transcription factors may depend on the cell type. HIF-α expression decreases upon prolonged exposure of cells to hypoxic conditions. Searches for genes that are upregulated upon prolonged hypoxia have been conducted, and matrix metalloprotease-1 (*MMP1*) was identified as one such gene [36]. MMP1 is a collagenase that is involved in extracellular matrix remodeling and promotes invasion of various cell types such as fibroblasts, endothelial cells (ECs), and cancer cells [37]. MMP1 is upregulated in HeLa cells under hypoxic conditions for 24 to 48 h, whereas HIF-1α starts to be downregulated. Analysis of the MMP1 promoter region identified sequences that are similar to the NF-κB- and CREB-binding sites. Importantly, this promoter is activated upon prolonged hypoxia, and inhibition of NF-κB and/or CREB significantly reduces its activity in response to hypoxia. Under hypoxic conditions, NF-κB is activated in several different ways. HIF-1 likely plays a role in activation of NF-κB because it induces expression of NF-κB and is involved in survival of neutrophils [35]. Furthermore, PHD is also involved in activation of NF-κB. PHD hydroxylates IκB and inhibits phosphorylation and degradation of IκB, which is required for activation of NF-κB [38]. Inhibition of PHD activity using inhibitors or siRNA-mediated knockdown (KD) enhances NF-κB activity upon TNF-α treatment, suggesting that PHD is a negative regulator of NF-κB. NF-κB is activated under hypoxic conditions, while PHD is inhibited; therefore, PHD is likely involved in hypoxic activation of NF-κB. KO of PHD1 increases expression of anti-apoptotic genes in hepatocytes, leading to a reduction in the cell death rate [39]. Moreover, NF-κB p65 is SUMOylated upon hypoxic treatment in hepatocellular carcinoma. KD of SUMO1 decreases NF-κB activity and reduces the growth and motility of hepatoma cells [40]. Furthermore, PHD2 is downregulated in certain tumors and this increases expression of *IL-8* and *angiogenin* genes, which is regulated by NF-κB [41]. Hypoxia induces NF-κB activation in different tissues and cancers. Human pulmonary microvascular ECs play a prominent role in the development of pulmonary artery hypertension (PAH). Upon exposure of ECs to hypoxia, NF-κB is activated and expression of *Endothelin1* and *ICAM1* increases [42]. Targeting of RelA in ECs reduces smooth muscle cell proliferation and inflammation associated with PAH. COMMD1 is a negative regulator of NF-κB and is downregulated under hypoxic conditions during osteoclastogenesis [43]. Hypoxic treatment induces expression of TRIM29, which is a tumor suppressor in breast cancers [44]. Expression of TRIM29 is mediated by ATM, HIF-1, and NF-κB, and inhibits upregulation of TWIST1.

A co-regulatory mechanism also exists between the NF-κB and HIF-1 pathways. There is an interaction between glioblastoma and astrocytes during the hypoxic response. Specifically, astrocytes produce CCL20 and trigger NF-κB activation, which results in upregulation of HIF-1α in glioblastoma [45]. Hypoxia and inflammation are prominent features of solid tumors. In oral squamous cell carcinoma, Toll-like receptor (TLR)3 and TLR4 activate NF-κB and induce HIF-1α expression [46]. Furthermore, HIF-1 directly regulates *TLR3* and *TLR4* expression, thus establishing a positive regulatory loop between NF-κB and HIF-1.

### 2.3. CREB

CREB is another transcription factor that upregulates MMP1 expression upon prolonged hypoxia [36]. Originally, CREB was identified as a transcription factor that mediates gene expression downstream of cAMP signaling [47]. Phosphorylation of CREB is required for its transactivation. Serine 129 is one of the best-characterized phosphorylation sites in CREB and is phosphorylated by protein kinase A (PKA). CREB is also phosphorylated by protein kinase C, Akt, Ca^2+^/calmodulin-dependent kinase, and MAPK-activated protein kinase 2 independent of the typical cAMP-PKA-CREB signaling axis [48]. We demonstrated that phosphorylation of CREB at serine 129 occurs upon prolonged hypoxia in several cancer cell lines, including HeLa and MCF7, although the kinase responsible has not been identified [36]. Transcriptional activity of CREB is upregulated along with an increase in its phosphorylation. The mechanism by which phosphorylation of CREB is induced upon chronic, but not acute, hypoxia remains to be clarified. One important role of CREB during prolonged hypoxia is to regulate the migration and invasion of cancer cells. Prolonged hypoxia induces phosphorylation of CREB and increases expression of genes involved in cell migration [36]. Accordingly, KD of CREB in breast cancer MDA-MB231 cells significantly decreases the number of metastatic nodules formed in the lung. Moreover, CREB is SUMOylated upon prolonged hypoxia [49]. SUMOylation stabilizes CREB and increases its activity, leading to induction of the EGFR ligand amphiregulin, which induces proliferation of epithelial cells [50]. CREB is also activated upon endoplasmic reticulum stress [51]. When cells are simultaneously exposed to hypoxia and deprived of nutrients, CREB is strongly activated as early as 6 h, indicating that severe stress promotes activation of a CREB kinase(s). Genes belonging to the Gene Ontology groups “cell–cell adhesion”, “extracellular matrix organization”, and “angiogenesis” were identified as possible targets of CREB by comparing wild-type and CREB-KD cells. These gene groups overlap with biological responses to hypoxia. CREB has been implicated to play a role not only in cancer, but also in other hypoxia-related diseases. In pulmonary arterial ECs exposed to hypoxic conditions, PAH signaling is recapitulated, Nox1 expression is induced, and reactive oxygen species are produced, resulting in activation of CREB. CREB induces Gremlin1 and promotes PAH symptoms [52]. The hypoxic response is important for survival of both neurons and astrocytes in ischemic diseases. HIF-1 and CREB are activated in astrocytes upon hypoxic treatment, and PKA plays a key role in the activation of both molecules [53]. CREB also functions in iron metabolism by regulating IRP1 [54]. Hypoxic activation of CREB induces IRP1 expression via the PI3K-Akt pathway. CREB also cross-reacts with HER-2/neu signaling in HER-2/neu-overexpressing cancers [55]. Hypoxia activates CREB and induces GLUT1 and VEGF in such cells, and this is inhibited by HER-2/neu signaling inhibitors. Interestingly, the same report also indicates that CREB translocates into mitochondria and regulates their metabolism, which is independent from its well-established function as a nuclear transcription factor.

### 2.4. Roles of other Transcription Factors in Hypoxia

The activities of other transcription factors also change under hypoxic conditions. Various transcription factors that play major roles in the oxidative stress response, immune responses, and cell proliferation, such as NF-E2-related factor-2 (Nrf2), signal transducers and activators of transcription (STATs), and Myc, will be described in the context of hypoxia in this section.

(a) Nrf2

Nrf2 is a transcription factor that regulates expression of anti-oxidant genes. This molecule is stabilized and activated in response to electrophiles [56]. Hypoxic conditions also induce expression and activity of Nrf2 when reactive oxygen species are produced. Stabilization of HIF-1α and expression of its target genes are reduced in Nrf2-KD cells under hypoxic conditions, indicating that Nrf2 plays a role in regulation of HIF-1α expression [57,58]. In addition, siRNA-mediated depletion of HIF-1α reduces expression of Nrf2 in C2C12 cells, suggesting that a co-regulatory mechanism exists between these two molecules [59]. By contrast, Nrf2 activation peaks after 24 h, while the HIF-1 pathway is activated after 72 h in an animal model of cerebral ischemia/reperfusion, indicating that these two factors function separately upon ischemia-reperfusion injury [60]. Hypoxia-dependent chemotherapeutic resistance is mediated by the catalytic and modifier subunits of glutamate-cysteine ligase, which are upregulated by Nrf2 in breast cancer cells [61].

(b) STATs

STATs comprise a family of transcription factors that are activated by cytokine signaling [62]. These molecules are phosphorylated and activated by JAK kinases. STAT3 is constitutively activated in certain types of cancer cells [63]. The level of phosphorylated STAT3 is increased in the hypoxic region of ovarian cancer [64]. Activation of STAT3 promotes cell survival and growth, whereas treatment with a STAT3 inhibitor reduces the level of STAT3 and expression of its target genes, such as *Bcl-xL*, *cyclin D2*, and *VEGF*. Hypoxic conditions induce expression and/or serine phosphorylation of STAT1, STAT3, and STAT5 in breast cancer MCF7 cells [65]. In hypoxic breast cancer, production of *EPO* is induced and this activates the JAK-STAT pathway to promote tumorigenesis and self-renewal of tumor-initiating cells [66]. Ganetespib, a Hsp90 inhibitor, downregulates *PDGFA*, *FGF2*, *Ang-1*, *Ang-2*, *TGFβ1*, *VEGF*, *HIF-1*α, and *STAT3* in human colorectal cancer cell lines, and has been shown to inhibit the growth of tumor xenografts [67]. Chronic hypoxic culture of LNCaP cells for more than 6 months activates the JAK/STAT pathway together with the Akt and HIF-1 pathways, and induces expression of matrix metalloproteinases [68]. Hypoxic treatment also activates STATs in other cell types. In rheumatoid arthritis synovial fibroblasts, hypoxic treatment activates HIF-1, STAT1, and STAT3 [69]. Hypoxic treatment induces cell invasion, migration, and cytokine production, which are inhibited by siRNA-mediated depletion of STAT3. STAT3 is activated in pulmonary vascular smooth muscle cells upon hypoxic treatment [70]. This induces proliferation of these cells, which is counteracted by overexpression of SOCS3.

(c) Myc

Myc is a transcription factor that induces expression of a wide range of genes related to regulation of cell growth, differentiation, and death. Myc also induces expression of genes involved in angiogenesis and metabolism [71,72]. HIF-1α and Myc interact in cells, and this inactivates Myc; thus HIF-1α is a negative regulator of Myc [73]. Overexpression of Myc promotes cell cycle progression even under hypoxia, but also increases expression of proapoptotic genes such as *NOXA* and *PUMA*, which enhances cell death [74]. Formation of VHL-deficient renal clear cell carcinoma is promoted by HIF-2α and inhibited by HIF-1α. Interestingly, in contrast with HIF-1α, HIF-2α enhances Myc activity by promoting binding of Myc to its co-activators and thereby induces expression of its target genes [75]. A study reported that Myc activity is required for the normal hypoxic response and hypoxia-dependent glycolytic reprogramming in glioblastoma [76]. Thus, Myc and HIF-1 cooperate in response to hypoxic conditions in certain cell types. Furthermore, Myc suppresses expression of a long noncoding RNA called IDH-AS1, which induces IDH1 enzymatic activity and downregulates HIF-1α due to an increase in α-KG [77]. In this context, Myc is a positive regulator of HIF.

## 3. Post-Transcriptional Regulation under Hypoxia

### 3.1. Pre-mRNA Splicing and Splicing Regulators

**Figure 3:**
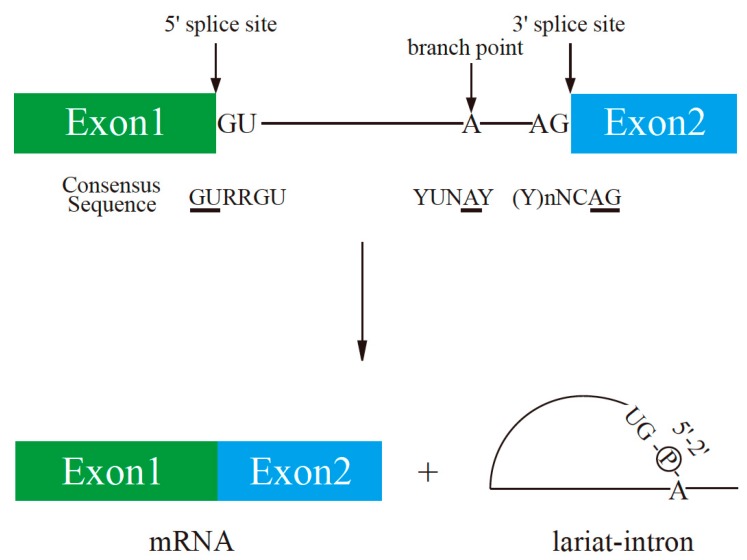
Schematic representation of the splicing reaction and essential splicing signals. Conserved sequence elements of metazoan pre-mRNAs are indicated. R and Y stand for purine and pyrimidine residues, respectively. N indicates any nucleotide. The adenosine residue used as a branch nucleotide is underlined.

**Figure 4:**
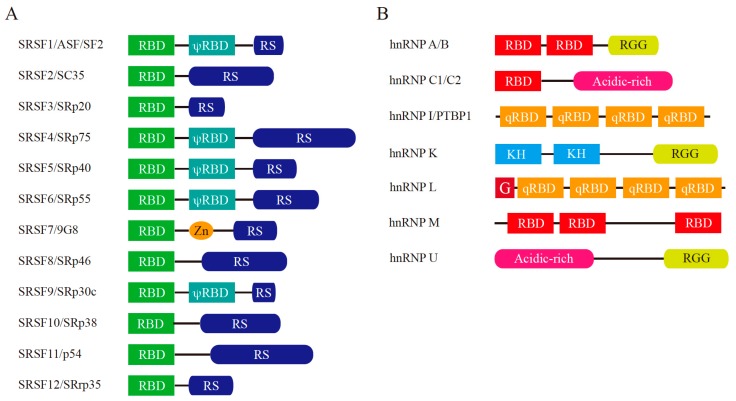
Schematic representation of major splicing regulators: serine-arginine-rich (SR) proteins and heterogeneous nuclear ribonucleoproteins (hnRNPs). (**A**) Structure of human SR proteins. Current serine-arginine-rich splicing factor (SRSF) names are shown on the left together with the protein aliases. RBD: RNA-binding domain (also known as RNA recognition motif (RRM)), RS: arginine-serine dipeptide repeat-rich region, Zn: Zn-binding domain, ψRBD: RBD homology domain. (**B**) Structure of human hnRNPs. RBD: RNA-binding domain, RGG: arginine-glycine-glycine repeat-rich region, Acidic-rich: acidic amino acid residue-rich region, qRBD: non-canonical RBD, G: Glycine rich region, KH: K homology domain.

In addition to transcriptional regulation mediated by HIFs and other transcription factors, as described in the first part of this review, post-transcriptional regulation also occurs in response to hypoxia. In higher eukaryotes, most genes are interrupted by intervening sequences called introns. After transcription, the primary transcripts, termed precursor mRNAs (pre-mRNAs), undergo many processing steps such as pre-mRNA splicing, polyadenylation, and editing. Pre-mRNA splicing removes introns from pre-mRNAs and ligates the remaining regions, termed exons. This is called constitutive splicing, in which all exons are ligated in order without any nucleotide deletions or insertions. The essential signals for the splicing reaction reside at both ends of introns (Figure 3) [78]. There is a consensus sequence of GURRGU (R stands for purine) at the 5′ end of introns in humans. The first G nucleotide is the cleaved site for the first step of the splicing reaction. A CAG consensus sequence is often present at the 3′ end of introns (Figure 3). In addition, a stretch of pyrimidine residues is located 20 to 30 nucleotides upstream of the 3′ splice site and supports recognition of this site (Figure 3). The excised introns form a lariat structure, in which the G nucleotide in the 5′ splice site is conjugated with a branch point nucleotide by 2′–5′ phosphodiester formation (Figure 3). In addition to intronic splicing signals, many regulatory elements have been identified in exons that modulate splicing by enhancing or inhibiting exon recognition, such as exonic splicing enhancer (ESE) and exonic splicing silencer (ESS), respectively [79]. ESE enhances recognition of the exon in which it resides. On the other hand, ESS inhibits recognition of the exon in which it is located. Alternative splicing (AS) is another mode of pre-mRNA splicing [80,81,82]. There are several types of AS, such as cassette exon type, alternative 5′ or 3′ splice site usage, mutually exclusive exons, and intron retention [80,81,82]. AS contributes at least in part to the diversity of protein expression from the limited number of genes in eukaryotes. Moreover, AS is regulated by additional splicing regulators that interact with the spliceosome [80,81,82]. Among these regulators, one of the best-characterized factors is serine-arginine-rich (SR) protein [81,83,84]. SR proteins all contain one or two RNA-binding domains (RBDs) and an arginine-serine-rich (RS) domain at the amino-terminus and carboxy-terminus, respectively [81,83,84]. In humans, 12 SR proteins have been identified (Figure 4A). SR proteins bind to ESE and facilitate exon recognition by bridging the two sides of the exon via interacting with the U2AF heterodimer and U170K proteins through their RS domain [85]. Heterogeneous ribonucleoproteins (hnRNPs) are another group of well-characterized splicing regulators. hnRNPs are abundant nuclear proteins involved in many cellular functions, such as transcription, splicing, RNA transport/localization, translation, and RNA stability [86]. The hnRNP family comprises 20 proteins named A1–U, which harbor many different types of RBDs (Figure 4B) [86]. In contrast with SR proteins, some hnRNPs bind to ESS [86]. hnRNP A1 mediates exon skipping by binding to a high-affinity binding site in an exon that promotes the association of hnRNP A1 with regions upstream and downstream of the exon [87]. This inhibits binding of SR proteins to ESE and covering splice sites.

### 3.2. Pre-mRNA Splicing in the Hypoxic Response

AS is a regulatory platform that allows tissue- and stage-specific expression. Recent studies demonstrated that pre-mRNA splicing plays an important role in the adaptation of cells to hypoxic conditions [88,89]. For example, AS plays an important regulatory role in angiogenesis. In response to hypoxia, tumors secrete the key ligand, vascular endothelial growth factor A (VEGFA), to promote the formation of new blood vessels. VEGFA is alternatively spliced, the most abundant isoform produced by use of alternative 3′ splice site is VEGF165b, which is an inhibitor of angiogenesis and distributed throughout most adult tissues. [90,91]. In contrast, this isoform was undetectable in most tumors. Down-regulation of VEGF165b was also observed in several cancer types, including prostate cancer and malignant melanoma [92,93]. Hypoxia-dependent AS was also reported in mouse cornea cells, in which inhibitory PAS domain protein (IPAS) mRNA is generated by AS of the HIF-3α locus [89,94,95]. The IPAS mRNA species contains the third unique exon 4a. In addition, a mechanism involving alternative 3′ splice site selection leads to a 14-nucleotide 5′ deletion of exon 3 and an 87-nucleotide deletion of exon 6 from the 3′ end. This mechanism, together with utilization of exon 4a, leads to production of mRNA encoding the IPAS protein. IPAS protein does not interact with HIF-β, but can bind to HIF-1α. Consequently, IPAS inhibits HIF-1-mediated activation of transcription [94,95]. A novel hypoxia-inducible splicing variant of the mouse *HIF-3α* gene was subsequently identified, which is predominantly expressed during the embryonic and neonatal stages. The first exon of HIF-3α pre-mRNA is replaced by the first exon of IPAS in this variant, which is named NEPAS (neonatal and embryonic PAS) [96]. In humans, HIF-1α pre-mRNA contains 15 exons. Nine HIF-1α mRNA isoforms are generated by alternative pre-mRNA splicing in human cell lines. One such isoform, HIF-1α516, is a dominant negative regulator of HIF-1α in vitro. HIF-1α516 protein competes with endogenous HIF-1α and suppresses HIF-1 activity, resulting in downregulation of hypoxia-inducible gene expression. This isoform has been suggested to act in a negative feedback loop and to preserve the balance between normoxic and hypoxic metabolism [97,98]. The human *HIF-3α* gene contains 19 exons and undergoes extensive AS, resulting in production of eight splicing isoforms [99]. All human HIF-3α protein variants can interact with HIF-β, HIF-1α, and HIF-2α [100]. Production of the HIF-3α4 isoform is upregulated in hypoxia, and this isoform directly binds to HIF-1α protein. HIF-3α4 suppresses HRE-dependent transcription, similar to IPAS in mice [94,101]. The fact that both HIF-1α and HIF-3α pre-mRNAs undergo extensive AS in human cells indicates that the hypoxic response mediated by HIFs is regulated at both the transcriptional and post-transcriptional levels. Following identification of hypoxia-dependent IPAS, other examples of hypoxia-dependent AS were reported. In human umbilical vein ECs, exon array analysis revealed that 342 exons are subject to AS under hypoxia-mimicking conditions [102]. Another report using a human liver cell line demonstrated that hypoxia causes 3059 AS events in 2005 genes [103]. Some of these are associated with tumors. Indeed, RNA sequencing analysis using a next-generation sequencing method identified ~2000 significantly regulated AS events during both acute and chronic hypoxia in MCF7 cells [104]. Accumulating evidence suggests that pre-mRNA splicing plays an important role in adaptation of tumor cells to hypoxic conditions, which enhances their proliferation and survival, by changing gene expression patterns [88,89].

### 3.3. Splicing Regulators in the Hypoxic Response

While several genes have been reported to undergo AS under hypoxia, only a few studies have addressed the underlying molecular mechanisms [103]. The molecular mechanism responsible for hypoxia-dependent AS of IPAS in HeLa cells was investigated using in vitro splicing assays. This revealed that expression of Cdc2-like kinase 1 (CLK1) increases under hypoxia in a HIF-1-regulated manner. HIF-1 increases mRNA expression and consequently protein expression of CLK1 in hypoxic cells. CLK1 hyper-phosphorylates SR proteins, leading to hypoxia-dependent AS patterns [105]. SR proteins comprise a highly conserved family of splicing factors that are expressed throughout metazoans and play diverse roles in both constitutive splicing and AS [81,83,84]. The C-terminal RS domain of SR proteins is highly phosphorylated, and phosphorylation of this domain affects the interactions of SR proteins with other proteins and RNAs as well as their intracellular localizations [106]. Hyper-phosphorylation of SR proteins alters the specificity of their RNA interactions in hypoxic cells. SR proteins interact with specific RNA sequences under hypoxia, which they do not bind under normoxia. This leads to production of mRNAs that encode proteins required for adaptation of cells to hypoxic conditions [105]. A recent study of prostate cancer PC3 cells demonstrated that hypoxia alters many AS patterns of genes and that expression of SRSF1, SRSF2, SRSF3, the SR protein kinase CLK1, and SR-specific protein kinase 1 (SRPK1) is significantly increased in hypoxia [107]. Taken together, it is highly likely that elevated expression of SR proteins and SR protein kinases facilitates adaptation of cells to hypoxic conditions via AS of key cancer-associated genes.

## 4. Conclusions

Cellular responses to hypoxia are complicated because they involve both transcriptional and post-transcriptional steps. It is well accepted that HIFs and other transcription factors are critical for the hypoxic response. Moreover, accumulating evidence suggests that pre-mRNA splicing plays important roles in gene expression under hypoxia. In addition to IPAS, many genes have been reported to undergo hypoxia-dependent AS [102,103,104,105,107,108]. However, the molecular mechanisms that regulate oxygen tension-dependent AS remain unclear. In addition to SR proteins, hnRNPs, which constitute a large protein family, may be involved in hypoxia-dependent AS. Further studies are required to elucidate these mechanisms by identifying cis-acting elements in pre-mRNAs and trans-acting splicing regulators. In addition, interplay between transcription and splicing in hypoxic cells must be explored. These investigations are essential to comprehensively understand the cellular hypoxic response and will likely increase our knowledge of gene expression regulation and adaptation in tumors. We sincerely hope that these analyses will help to identify new targets and possible treatments for cancers.
